# Identification of Flavonoid C-Glycosides as Promising Antidiabetics Targeting Protein Tyrosine Phosphatase 1B

**DOI:** 10.1155/2022/6233217

**Published:** 2022-06-24

**Authors:** Athika Rampadarath, Fatai Oladunni Balogun, Charlene Pillay, Saheed Sabiu

**Affiliations:** Department of Biotechnology and Food Science, Faculty of Applied Sciences, Durban University of Technology, P.O. Box 1334, Durban 4000, South Africa

## Abstract

Protein tyrosine phosphatase 1B (PTP1B), a negative regulator of the insulin signaling pathway, has gained attention as a validated druggable target in the management of type 2 diabetes mellitus (T2DM). The lack of clinically approved PTP1B inhibitors has continued to prompt research in plant-derived therapeutics possibly due to their relatively lesser toxicity profiles. Flavonoid C-glycosides are one of the plant-derived metabolites gaining increased relevance as antidiabetic agents, but their possible mechanism of action remains largely unknown. This study investigates the antidiabetic potential of flavonoid C-glycosides against PTP1B *in silico* and *in vitro*. Of the seven flavonoid C-glycosides docked against the enzyme, three compounds (apigenin, vitexin, and orientin) had the best affinity for the enzyme with a binding score of –7.3 kcal/mol each, relative to –7.4 kcal/mol for the reference standard, ursolic acid. A further probe (in terms of stability, flexibility, and compactness) of the complexes over a molecular dynamics time study of 100 ns for the three compounds suggested orientin as the most outstanding inhibitor of PTP1B owing to its overall -34.47 kcal/mol binding energy score compared to ursolic acid (-19.24 kcal/mol). This observation was in accordance with the *in vitro* evaluation result, where orientin had a half maximal inhibitory concentration (IC_50_) of 0.18 mg/ml relative to 0.13 mg/ml for the reference standard. The kinetics of inhibition of PTP1B by orientin was mixed-type with *V*_max_ and *K*_*m*_ values of 0.004 *μ*M/s and 0.515 *μ*M. Put together, the results suggest orientin as a potential PTP1B inhibitor and could therefore be further explored in the management T2DM as a promising therapeutic agent.

## 1. Introduction

The global occurrence of type 2 diabetes mellitus (a disease condition attributed to an increased level of glucose in the systemic circulation arising from insulin insensitivity, inaction, or both) is rapidly becoming a concerning healthcare issue due to its associated mortality, morbidity, and economic burden [1–3]. Currently, more than 463 million individuals worldwide are diabetic, and the accompanying annual mortality stood at 1.5 million [4]. Since insulin deficiency and/or resistance is a direct consequence of increased glycaemic index, mechanism geared towards optimal regulation of the hiked systemic glucose concentration is not only germane in the effort towards management of the T2DM but imperative, and the protein tyrosine phosphatase (PTP) family represents one of the viable therapeutic targets in this context [5].

Protein tyrosine phosphatase 1 B (PTP1B) is a nontransmembrane protein of the PTP family [6] and functions primarily to balance phosphorylation/dephosphorylation of tyrosine, a mechanism required to ensure cell proliferation and differentiation in mammals [7]. Additionally, it serves as a downregulator of the insulin and leptin signalling pathway, catalysing the dephosphorylation of insulin receptor (IR) and insulin receptor substrate (IRS) which contributes to the pathogenesis of T2DM and obesity by the induction of insulin and leptin resistance, respectively [8]. In fact, tyrosine phosphorylation triggers IRS attachment to phosphatidylinositol-3-kinase resulting in the formation of other secondary intermediates, thus activating Akt signalling pathway, and when this happens, glucose transport 4 (GLUT4) is catapulted into the cell membrane (from vesicle of the cytoplasm) enhancing glucose transport [9, 10]. Hence, PTP1B modulation may come in handy as a druggable target in T2DM and has been exploited for new inhibitors or drugs in the management of T2DM [11].

Flavonoids are one of the major plant secondary metabolites and constitute the largest group of phenolics finding therapeutic applications against several degenerative diseases including T2DM [12–18]. Generally, flavonoids exist as either O- or C-glycosides with flavone and flavonol as the most abundant in nature [5, 13, 19]. Flavonoid C- and O-glycosides such as luteolin, apigenin, hesperetin, and naringenin have been previously reported as potential PTP1B inhibitors [5, 14, 20]. While the economic importance of flavonoids and their related compounds, such as orientin, vitexin, isoorientin, and apigenin, is far-reaching and in fact, speculated to be reaching 1.2 billion US dollars by 2024 [21], the great need for more studies on the effectiveness of these compounds on diabetes-related enzymes especially PTP1B for effective management of T2DM is important. In fact, flavonoid C-glycosides such as orientin and hesperetin have found therapeutic applications in the management of ailments such as hypertension, inflammation, cancer, and Alzheimer, identified as risk factors of T2DM [5, 20, 22–27]. Although some flavonoid C-glycosides have been previously evaluated as PTP1B inhibitors [14, 20], the exact mechanism of molecular interactions between the compounds and the amino acid residues at the binding pockets of PTP1B is still lacking to date. Here, we investigated the flavonoid C-glycosides (apigenin, aspalathin, isoorientin, isovitexin, puerarin, vitexin, orientin) against PTPIB as a druggable target in T2DM with a view to establishing their probable mechanism of antidiabetic action using molecular dynamics (MD) simulation and *in vitro* experimental models.

## 2. Materials and Methods

### 2.1. Chemicals and Reagents

The ursolic acid, orientin, para-nitrophenylphosphate (*p*-NPP), PTP1B (human recombinant), ethylenediaminetetraacetic acid (EDTA), and dithiothreitol (DTT) used were obtained from South Africa branch of Sigma-Aldrich. The use of analytical grades of unlisted chemicals and reagents was ensured.

### 2.2. In Silico Analysis

PTP1B X-ray crystal structure (PDB ID: 2HNQ) was accessed (https://www.rcsb.org/) from RSCB Protein Data Bank. The optimization and preparation of PTP1B structure via UCSF Chimera V1.14 according to Branden et al. [28] protocol involve eliminating the missing side chain or backbone atoms, protein residue connectivity, water molecules, and nonstandard naming. PubChem (https://pubchem.ncbi.nlm.nih.gov/) was used to retrieve the 3D structures of the studied flavonoid C-glycosides (apigenin, aspalathin, isoorientin, isovitexin, puerarin, vitexin, orientin) and reference standard, ursolic acid [14]. The optimization (Gasteiger charges and nonpolar hydrogen atom addition) of the retrieved 3D structures was achieved using the Avogadro software v2.0 as described by Kim et al. [29]. Subsequently, the optimized flavonoids (ligands) and PTP1B (receptor) were made to undergo the process of molecular docking.

The docking of the optimized flavonoids and ursolic acid into the active site of PTP1B was executed by AutoDock Vina Plugin on Chimera V1.14 upon the identification of the PTP1B binding sites [30]. The grid box with generated *x*-*y*-*z* coordinates was mounted on the binding site, and the optimized flavonoids and standard were docked. Judging by the docking scores, the resulting docked complexes with the best pose for each compound were selected and subjected to MD simulation.

The MD simulation was carried as described by Sabiu et al. [17] on the graphical processing unit (GPU) version provided with the AMBER 18 application where the description or features of the system were made possible by the AMBER force field (FF18SB variant) [31]. With the aid of restrained electrostatic potential (RESP) and the general AMBER force field (GAFF) protocols, the compounds' partial atomic charges were obtained with ANTECHAMBER. Hydrogen atom (H^+^) addition, Na^+^, and Cl^−^ counter ions (for system neutralization) inclusion were made possible by the Link, Edit, and Print (LEaP) program or module of AMBER. This was followed by the numbering of the enzyme amino acid residues and suspension of each system in an orthorhombic box of TIP3P filled with H_2_O molecules to ensure that all atoms lie 8 Å of all edges of the box.

The system began with 2000 minimization steps, restricted with 500 kcal/mol potential for both solutes on another 1000 steps with the aid of the steepest descent procedure and subsequently with conjugate gradients of 1000 steps. Additionally, full minimization stage follows with 1000 steps without restraint using conjugate gradient algorithm. The next MDS stage is heating carried out 50 ps, beginning from zero kelvin (0 K) to 300 K, to ensure a constant volume of water and atoms. Thereafter, the solutes were subjected to potential harmonic restraint (10 kcal/mol) and collision frequency (1.0 ps). Equilibration stage follows equilibrating the system with 500 ps while keeping constant the heating temperature at 300 K. In order to mimic an isobaric-isothermal ensemble (NPT), other parameters including several atoms and pressure (at 1 bar) were maintained still with the latter measured with Berendsen barostat [32].

While MD simulation overall time was 100 ns and each simulation employing SHAKE algorithm to contract H^+^ bonds, the step size with aid of SPFP precision model was 2 fs. The simulations coincided with NPT, constant pressure (1 bar), randomized seeding, temperature (300 K), pressure-coupling constant (2 ps), and Langevin thermostat with a collision frequency (1.0 ps) [32].

Subsequently, saving of system coordinates and analysis using PTRAJ of the trajectories at every 1 ps followed by the involvement of CPPTRAJ module (installed in AMBER) in the analyses of root mean square fluctuation (RMSF), root mean square deviation (RMSD), and radius of gyration (RoG).

Molecular mechanics/GB surface area method (MM/GBSA) was employed based on Ylilauri and Pentikäinen [33] for the estimation and comparison of the systems' binding affinity in an effort to calculate the free binding energy (Δ*G*). The average of Δ*G* over 100000 snapshots was obtained in 100 ns trajectory. The Δ*G* for individual molecular species (complex, ligand, and protein) was assessed with aid of the following five expressions (1)–(5): ∆*G*_bind_ = *G*_complex_ − *G*_receptor_ − *G*_ligand_ (1), ∆*G*_bind_ = *E*_*g*_ + *G*_*s*_ − *TS* (2), *E*_*g*_ = *E*_*i*_ + *E*_*v*_ + *E*_*e*_(3), *G*_*s*_ = *G*_*GB*_ + *G*_*SA*_(4), and *G*_*SA*_ = *γSASA*(5), where the term *E*_*g*_ refer to the energy of the gas-phase, consisting of internal energy (*E*_*i*_), Coulomb energy (*E*_*e*_), and van der Waals energies (*E*_*v*_). FF14SB force field terms were employed in direct estimation of *E*_*g*_. Energy release from polar states (GGB) and nonpolar states (G) were adopted for the calculation of solvation free energy, *G*_*s*_. Solvent-accessible surface area (SASA) using a water probe radius (1.4 Å) was used in the determination of nonpolar solvation energy, GSA. In contrast, the polar solvation, GGB contribution was estimated by solving the GB equation. *T* and *S* represent temperature and solute total entropy, respectively.

### 2.3. In Vitro PTP1B Inhibitory Assay

Following the consideration of results from the *in silico* evaluation, orientin had the highest negative binding energy value post-MD simulation and was selected for the *in vitro* analysis. The inhibitory activity of orientin was determined by the hydrolysis of *p*-NPP to *p*-nitrophenol (*p*-NP), using the method of Rocha et al. [34] with modifications. Twenty microliters PTP1B (0.2 units/ml) was added to the wells of the 96 well microtiter plate and diluted with a PTP1B reaction buffer containing 50 mM citrate (pH of 6), 0.1 M sodium chloride, 1 mM EDTA, and 1 mM of dithiothreitol (DTT). Thereafter, 40 *μ*l of varying concentrations (0.031-0.5 mg/ml) of orientin was added to the wells and incubated at 37°C for 10 min, followed by 40 *μ*l addition of 2 mM *p*-NPP (in PTP1B buffer solution) and a further incubation for 20 min at 37°C. The resulting reaction medium was then terminated by the addition of 10 M sodium hydroxide, and the amount of *p*-NP produced through the dephosphorylation of *p*-NPP was estimated spectrophotometrically 405 nm. Ursolic acid was similarly treated as orientin and employed as a reference standard with all experiments conducted in triplicates. The percentage inhibition of PTP1B by orientin and ursolic was determined by the expression, Abs_0_ − Abs_1_/Abs_0_ × 100 (where Abs_0_ is the absorbance of the blank and Abs_1_ is the absorbance of either orientin or ursolic acid) before determination of their half-maximal inhibitory concentrations (IC_50_) using nonlinear dose-response curves.

### 2.4. Enzyme Kinetics Analysis

The mode of inhibition of PTP1B by orientin was determined as described by Jung et al. [35]. In brief, orientin (IC_50_ value) was tested against varying concentrations (1, 2, 3, 4, and 5 mM) of *p-*NPP. Twenty microliters each of the PTP1B and orientin was added in one set occupying 5 wells of a 96-well microtiter plate and incubated for 10 min at 37°C. Similarly, PTP1B and buffer were added in another set of 5 wells. Thereafter, different concentrations (1, 2, 3, 4, and 5 mM) of the *p-*NPP were added to the two sets of wells containing the reaction mixtures and incubated at 37°C for 20 min before absorbance measurement at 405 nm. The concentration of *p-*NP produced was determined from the calibration curve of *p*-NPP, and the amount of product over time was thereafter calculated and converted to reaction rates. The mode of inhibition was subsequently determined by plotting the graph of inverse of reaction rate (1/V) against the inverse of substrate concentration (1/[S]) from where the *V*_max_, *K*_*m*_, and *K*_cat_ values were derived [36].

### 2.5. Pharmacokinetic Attribute Prediction

The pharmacokinetic properties including the ADME features and drug-likeness of orientin are predicted using the SWISS ADME server [37].

### 2.6. Statistical Analysis

For the *in vitro* experiments, data analyses were carried out by one-way analysis of variance (ANOVA), followed by Bonferroni's multiple comparison test using GraphPad Prism version 3.0 for Windows (GraphPad Software, San Diego, California, USA), and results are expressed as mean ± standard error (SEM). However, except otherwise stated, the Origin data analysis software V18 was adopted on the raw data plots for the *in silico* evaluations [38].

## 3. Results and Discussions

### 3.1. In Silico Assessment

Of the flavonoid C-glycosides evaluated in this study, orientin, vitexin, and apigenin had the highest docking score of -7.3 kcal/mol each in relation to -7.4 kcal/mol obtained for the reference standard, ursolic acid (Table 1). Also, the results of the thermodynamics evaluation during the 100 ns MD simulation revealed that the PTP1B-orientin complex had the highest negative binding energy (-34.47 kcal/mol) followed by vitexin (-33.67 kcal/mol) and apigenin (-24.58 kcal/mol) complexes, respectively (Table 2). These binding energy values for the study compounds were greater than that of ursolic acid (19.24 kcal/mol) (Table 2).

Molecular docking is an effective method aimed at determining the interaction and the mode of binding of compounds to the active site of the enzyme [19]. It depicts the competence of the ligand at the enzyme catalytic site [17], and a compound or ligand with the highest negative binding score is identified as having the best affinity for the enzyme [39]. This can be seen with vitexin, orientin, and apigenin revealing the most negative score (-7.3 kcal/mol) for PTP1B in this study which was at par with ursolic acid (-7.4 kcal/mol), the standard drug. Thus, suggesting the likelihood of the compounds having good interaction with PTP1B, this is consistent with previous studies on molecular docking of compounds [40]. However, there are reports or studies where flavonoids such as quercetin, morin, isorhamnetin, vitexin, isoorientin, and luteolin tend to be better posed with enzymes such as chymotrypsin-like protease, alpha-glucosidase, and PTP1B than standard inhibitors [20, 41–44]. However, due to limitations of molecular docking serving only as a preliminary analysis of a ligand's affinity for protein's binding pocket, MD simulation was performed over a -100 ns to account for the protein's behavior upon binding of the respective flavonoid glycosides, through better elucidation of the ligand's affinity by binding free energy, as well as vital conformational information regarding structural stability, flexibility, and compactness taken as post-MD simulation indices [45]. In this study, the higher binding free energies of the flavonoids especially orientin alluded to stronger affinity of these compounds (orientin, vitexin, and apigenin) for the active site of PTP1B than ursolic acid which on the long run would suggest better stability of the orientin-PTP1B, vitexin-PTP1B, and apigenin-PTP1B complexes as previously affirmed [46]. Higher binding energies (depicted by the most negative score) of related flavonoids such as naringenin and albanol B in complexation with PTP1B have been established [14, 42].

**Figure 1:**
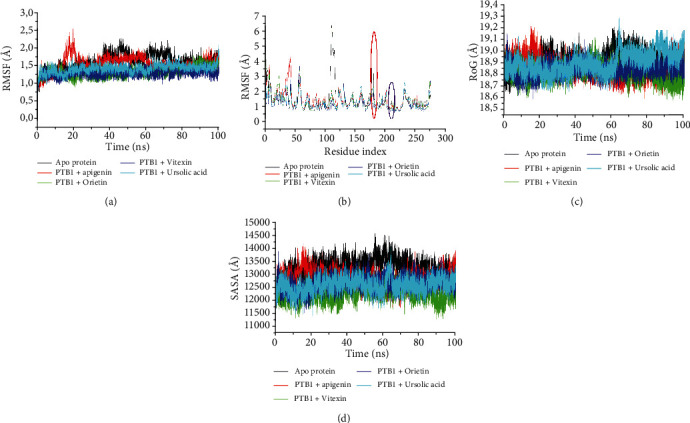
(a) Root mean square deviation (RMSD), (b) root mean square fluctuations (RMSF), (c) radius of gyration (RoG), and (d) solvent accessible surface area (SASA) plots of comparison between protein tyrosine phosphatase 1B (PTP1B) and flavonoid C-glycosides and ursolic acid determined over 100 ns molecular dynamics simulations. PTB1: PTP1B (protein tyrosine phosphatase 1B).

Molecular dynamics simulation has been adjudged as a viable *in silico* method through which dynamic data at atomic spatial resolution can be acquired [47]. It studies the conformational changes likely to occur due to the binding of the ligand to the enzyme which could affect the stability of the complex as well as the eventual biological activity of the enzyme [48]. Hence, understanding the stability, flexibility, and compactness of the enzyme-ligand complex is germane towards studying the interaction of the ligand at the binding pocket of the enzyme. In this study, the average RMSD value of the unbound PTP1B was 1.68 Å, which was higher than the values for the complexes formed with the study flavonoids and ursolic acid except the apigenin complex (1.70 Å) with comparable value to the apoenzyme (Table 3). The lowest average RMSD value was found with vitexin (1.12 Å), followed by orientin (1.32 Å) and ursolic acid (1.50 Å) (Table 3). Furthermore, it was observed that the test flavonoids and the reference standard converged at 15 ns and subsequently at 45 ns (Figure 1(a)). After 60 ns of evaluation, the protein-ligand complexes begin to converge and thereafter displayed similar equilibration throughout the simulation period (Figure 1(a)). Root mean square deviation is a function of structural rigidity of the ligand-protein complex which ultimately is a function of stability [40]. A lower average RMSD value of a resulting complex compared to that of the apoenzyme signifies better stability [17] and vice versa. In this study, the lowest average RMSD values of PTP1B complexation with both vitexin and orientin as well as ursolic acid compared with the unbound PTP1B are suggestive of better stability of the complexes. In fact, vitexin- and orientin-PTP1B complexes having the lowest values compared to ursolic acid complex are an indication that both vitexin and orientin conferred better stability on PTP1B than ursolic acid, signifying their potentials as possible lead moieties of therapeutic importance against PTP1B.

Regarding RMSF, fluctuations were observed throughout the simulation period with PTP1B as well as when the protein was complexed with the flavonoid glycosides (Figure 1(b)). However, a lesser degree of fluctuation was observed upon binding of the flavonoids in a similar manner as observed with RMSD, with vitexin-PTP1B complex having the lowest average RMSF value (0.98 Å) followed by orientin (0.99 Å), ursolic acid (1.05 Å), and apigenin (1.22 Å), and these values were lower than that of the unbound PTPIB (1.28 Å) (Table 3). It was noteworthy to see decreased fluctuations around the catalytically important regions (residues 175-188 and 214–221, representing the WPD loop (red circle) and the P-loop (purple circle; active site), respectively) of the protein, when in complexation with the flavonoid glycosides compared to the unbound PTP1B (Figure 1(b)). Similar to the observation with RMSD, the PTP1B-flavonoid complexes competed favorably with PTP1B-ursolic complex (Figure 1(b)). Root mean square fluctuation is a measure of flexibility of the ligand and the amino acid residue behavior at the binding pocket of the enzyme [17]. A high flexibility indicates high fluctuation (unstable bonds) and vice versa; a low flexibility or RMSF value is good because it means less distortion, hence a stable complex [43]. The ability of all the compounds (orientin, apigenin, vitexin) and ursolic acid to have lower RMSF values compared to the apoenzyme signalled lesser structural flexibility of the amino acid residues and thus a better flexibility for vitexin and orientin than ursolic acids, further suggesting their capability in stabilizing PTP1B upon binding better than ursolic acid. The WPD and P-loops play a role in the affinity of the inhibitor and identification of the tyrosine phosphorylation group of the substrates, respectively, and therefore, these regions must be critically considered while designing inhibitors against the PTP1B [49]. The P-loop is a rigid region of the PTP1B, and the binding of the flavonoid glycosides in this study appears to maintain that rigidity with negligible fluctuation which is an indication of good conformational behavior which in turn is indicative of enhanced stability of the complexes. Additionally, it was suggested by Liu et al. [50] that lower fluctuations at this active region could potentiate a decrease in the catalytic activity of the enzyme which favors a PTP1B inhibitor as was the case in this study. In accordance with one of the prerequisites of PTP1B inhibitors, the WPD loop (an established flexible region of PTP1B) must adopt a stable conformation upon the binding of an inhibitor. In this study, obvious fluctuations were observed with the unbound PTP1B but later regained its stability upon binding of apigenin, orientin, and vitexin, as seen with the decrease in mobility/fluctuation of the amino acids of the WPD loop. The protein subsequently adopted a more rigid and stable conformation, and the consequence of this change in the mobility of the loop is the prevention of substrate binding [51]. Jointly, the decreased fluctuations at the P-loop and WPD loop resulting in increased stability will present difficulty for the entry and binding of a substrate [45]. The decreased fluctuations around Val113-Ser118, Thr177-Pro185, and His214-Arg221 in this study correlate with a study conducted by Wu et al. [52] who suggest that decreased fluctuations of these amino acid residues enhanced stability around the active site of the enzyme upon the binding of a potent inhibitor. This was also emphasized by Barik et al. [53] that decrease in fluctuations around Tyr46, 111-121, WPD loop, and P-loop (upon binding of an inhibitor) is of importance for a potential PTP1B inhibitor, and this behavior was observed in the current study with both the study flavonoid glycosides and the standard complexes. Yan et al. [54] also suggest that these regions coupled with Tyr42 particularly interact strongly with potential PTP1B inhibitors, and this was equally observed with the interaction plots of the three flavonoid glycosides in this study. The results of the RMSF analysis in this study have provided vital information on the complexation of apigenin, orientin, and vitexin with PTP1B regarding its catalytically important residues and their role in PTP1B inhibitory activity that could be further explored for designing novel PTP1B inhibitors.

The radius of gyration (RoG) plot (Figure 1(c)) of the apoenzyme, the flavonoid glycosides, and the ursolic acid showed stability around 18.9–19.0 Å. The average RoG of orientin-PTP1B complex (18.85 Å) and vitexin-PTP1B complex (18.81 Å) was insignificantly lower than that of the unbound protein (18.92 Å) which was though at par with apigenin-PTP1B complex (18.92 Å) and ursolic acid (18.94 Å) (Table 3 and Figure 1(c)). Radius of gyration is a measure of compactness of the ligand-enzyme (complex) system [36, 44]. Increased RoG value means reduced compactness while lower RoG value indicates strong compactness and consequently mean enhanced stability [19, 43]. Going by results of this study, the RoG values for orientin and vitexin are relatively similar, and both had the lowest RoG score compared to the unbound PTP1B suggestive of the compactness and stability of their complexes. However, this cannot be said of ursolic acid whose RoG value was higher than that of the apoenzyme; meaning, the complex formed was less stable compared to the studied flavonoid C-glycosides, thus, corroborating other aspect of the study such as binding free energy score, RMSD, and RMSF on the potential attributes of orientin and vitexin as probable lead compounds.

Solvent accessible surface area plot between the study flavonoids, reference standard, and the unbound PTP1B is shown in Figure 1(d), and there was a measure of hydrophobicity around 18 and 48 ns of simulation. The average SASA values of the flavonoids and the standard were generally lower when compared to 13194.41 Å for the unbound PTP1B (Table 3). In similar manner with other postdynamic simulation parameters, the vitexin-PTP1B complex (12307.95 Å) had the lowest SASA value compared to orientin-PTP1B complex (12621.41 Å) and the ursolic acid complex (12613 Å) (Table 3). Unlike the RoG, SASA is a measure of hydrophobic/hydrophilic residues on exposure to solvent [55]. The degree of fluctuation of SASA value of the unbound form of a protein will depend on the physiochemical properties of the amino acid residue(s) that was rearranged/mutated [56]. While SASA is an important parameter in energy determination of biomolecules as well as the hydrophobic interactions within the amino acid residues (nonpolar) as a consequence of enzyme stability, a lower SASA value of the resulting complex compared to the apoenzyme is an indication of heightened solvent exposure of the nonpolar residues of the complex leading to better stability of the complex [57] which was the case with all the three flavonoid glycosides and ursolic acid in this study. These results agreed with those of RoG in which decreased SASA value is indicative of the stabilization of the folded protein conformation and confirms that binding of the flavonoid glycosides to PTP1B did do not result in the formed complexes undergoing unfavorable conformational changes such as expansion of the protein structure and protein unfolding. This observation is also consistent with those of Kumar et al. [58] that suggest that a decrease in SASA values suggests good conformational behavior (folded protein).

**Figure 2:**
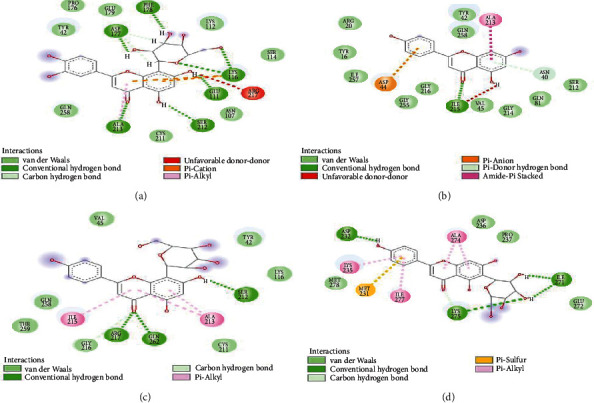
Interaction types and plots of (a) orientin, (b) apigenin, (c) vitexin, and (d) ursolic acid towards protein tyrosine phosphatase 1B (PTP1B) after 100 ns MD simulation.

The results obtained regarding the number and types of interactions formed in each complex post-MDS are presented in Figures 2(a)–2(d), while Table S1 and Figure S1 show the interactions during molecular docking. For the orientin-PTP1B complex, 15 interactions comprising six conventional hydrogen bonds with Phe178, Asp177, Lys116, Glu111, Ser212, and Ala213; eight van der Waal forces with Pro176, Gly179, Tyr42, Lys112, Ser114, Asn107, Cys211, and Gln258; and one unfavorable donor-donor bond (Arg217) were observed (Figure 2(a)). Orientin has same number of interactions with apigenin (though differs in type of bonds formed) consisting of 11 van der Waal forces (Arg20, Tyr16, Ile257, Gly255, Gly216, Val45, Gly214, Gln81, Ser212, Gln258, Tyr42), one conventional hydrogen bond (Ile215), *π*-anion (Asp44), *π*-donor H-bond (Asn40), and amide-*π* stacked (Ala213) (Figure 2(b)). Fewer numbers of interactions were found with vitexin containing 12 interactions (6 van der Waal (Val45, Gln258, Thr259, Cys211, Tyr42, Lys116), 3 conventional H-bond (Arg217, Gln262, Ser212), C-H (Gly216), and 2 *π*-alkyl (Ile215, Ala213)) (Figure 2(c)) and ursolic acid possessing the least number (11) of interactions (4 van der Waal forces (Asp236, Pro237, Met278, Glu272), 3 conventional H-bonds (Asp232, Lys275, Ile271), 3 *π*-alkyl (Lys235, Ile277, Ala274), and 1 *π*-sulphur bond (Met231)) (Figure 2(d)). A further observation regarding the overall hydrogen bonds revealed that the orientin-PTP1B complex had 41% which is in sharp contrast to 7, 23, and 31%, respectively, for the apigenin, vitexin, and ursolic acid complexes (Figures 2(a)–2(d)).

Interaction between binding the study flavonoids and the amino acid residues at the active site of PTP1B is represented by the ligand-enzyme interaction plots, and the type and number of interactions formed are crucial to the degree of affinity that will be established [59]. The interactions between orientin and PTP1B produced 15 interactions consisting of conventional hydrogen bonds, van der Waal forces, and unfavorable donor-donor bond. Though the interactions between apigenin and PTP1B also resulted in 15 interactions (conventional H-bond, van der Waal, *π*-anion, and amide-*π* stacked), however the presence of *π*-anion and amide-*π* stacked bonds as well as fewer number of H-bond (1) could be suggested as to why the binding energy score of apigenin was lower than that of orientin. The report from this study contradicted similar study from Ali et al. [20] against PTP1B (PDB ID: IT49) where though the number of H-bonds for apigenin and orientin is the same but the van der Waal forces are lesser. This is aside the fact that the interacting amino acid residues bear no resemblance as no MD simulation was performed in the study. The lowest number of bond interactions for ursolic acid as seen in this study is corroborated by the lower binding energy scores, though, this differs for vitexin as the fewer bond interactions shown did not correspond with binding energy score. More importantly, the implication of higher number of hydrogen bonds is a stronger and stable ligand-protein complex, which within the context of this study implies better inhibition. The higher the number of hydrogen bonds, the higher the residence time of the inhibitor at the catalytic site and thus an enhanced inhibitory activity [30]. This is equally true for the *π*-cation interaction being one of the strongest driving forces contributing to biological complexation process. Hence, besides other interactions, the higher percentage of hydrogen bond interactions in addition to the two *π*-cation interactions formed between orientin and PTP1B could have contributed to its enhanced stability which is also consistent with the results of the thermodynamic binding free energy in this study.

### 3.2. Pharmacokinetics Investigation

Analysis of the pharmacokinetics and drug-likeness features such as molecular weight (< 500 g/mol), number of hydrogen bond donor (not > 5), number of H-bond acceptor (< 10), bioavailability (> 10%), and octanol-water partition coefficient log *P* (not >5) showed that orientin had two violations exceeding the acceptable limit in the number of H-bond donor (8) and acceptor (11) while ursolic acid has one violation (Table 4). Druglikeness is a measure of similarity between certain compound and standard drug as evaluated based on molecular properties (such as hydrogen bonding, hydrophobicity, molecular weight, bioavailability, electron distribution, toxicity, and metabolic stability etc.) and structural merits [60, 61]. A vital index at predicting the worthiness of a compound as a drug candidate is Lipinski rule of five, which basically indicates that a compound will possibly exhibit poor bioavailability if it violates the five rules [62]. Consistent with this, the SwissADME software used in the study revealed or predicted two violations for orientin in the number of H-bond acceptor and/or donor exceeding the acceptable range while ursolic acid had a violation. However, in a report by Benet et al. [63], amendments or allowance had been made to Lipinski rule that in an effort towards drug development; two violations may be accepted for natural products or chemical derivatives of natural product developed for oral administration but not as injectables. Furthermore, a potential drug needs to be predicted to have at least 10% oral bioavailability [64], which favors orientin with a score of 17% and thus will be bioavailable orally.

### 3.3. In Vitro Evaluation

**Figure 3:**
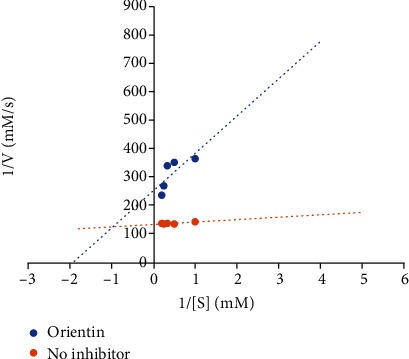
Lineweaver-Burk plot of orientin against PTP1B exhibiting mixed inhibitory mode.

Diabetes is a global health menace, and the prevalence of the disease which stand at 463 million as at 2019 continues to increase with heightened population [4]. It is projected that the number of sufferers by 2030 would be reaching 764 million [20]. Since inadequate or ineffective insulin plays a major role in the emergence of diabetes, disruption of insulin signalling pathway is germane to the pathogenesis of the disease. Protein tyrosine phosphatase negatively modulates the receptors of insulin in a cellular signal transduction pathway thereby causing insulin resistance; thus, inhibition of PTP1B would go a long way in the management of diabetes. Going by the results of the inhibitory effect of the test compounds, it was observed that ursolic acid and orientin inhibited the activity of PTP1B dose-dependently, with IC_50_ of ursolic acid (0.13 mg/ml) being significantly (*P* < 0.05) lower than that of orientin (0.18 mg/ml) (Table 5). A further probe into the kinetics of inhibition of PTP1B by orientin revealed a mixed mode (Figure 3) with a reduction in *V*_max_ from 0.005 to 0.004 *μ*M/s and corresponding increases in *K*_*m*_ and *K*_cat_ values from 0.048 to 0.515 *μ*M and 0.018 to 0.048 *μ*M/s for the control and orientin, respectively (Table 6 and Figure 3). Looking at the results of the *in vitro* evaluation, orientin dose-dependently inhibits the activity of PTP1B though the effect of ursolic acid was more pronounced than orientin, judging by the IC_50_ values, although it must be noted that the activity elicited by orientin against PTP1B fared well when compared with the activity depicted by orientin in a similar study by Ali et al. [20] where a higher IC_50_ value was reported. The mixed type of inhibition with accompanied changes in *V*_max_, *K*_*m*_, and *K*_cat_ is indicative of the binding of orientin to a site close to the active site as evidenced by the differences in the amino acid residue interactions formed during docking and post-MD simulation. This further underscores the significance of MD simulation in revealing the mechanism of molecular interactions between a ligand and a protein. While mixed-type inhibition combines competitive and uncompetitive inhibitions, the report of this study correlates to the work of Song et al. [65] who reported a mixed type of inhibition for all the geranylated flavonoids from *Paulownia tomentosa* against PTP1B. This suggests that orientin could either directly bind to the free PTP1B at a site close to its active site or bind to the PTP1B-substrate complex, the consequence of which the overall reaction rate will decrease, and the product will be produced at a slower rate. In contrast, ursolic acid has been reported to be a noncompetitive inhibitor by Rocha et al. [34]. Contradictorily, Ha et al. [66] reported ursolic acid to exhibit competitive inhibition against PTP1B. These differing results may be attributed to experimental conditions such as the time of the kinetic monitoring of the assay, differing enzyme, and substrate concentrations as well as the enzyme sequences (full length/catalytic domain) implemented in the respective study. However, these two differing submissions have been better established in this study with orientin eliciting a mixed inhibitory effect on PTP1B, and this is advantageous for orientin as its binding at a different site aside from the active site suggests higher specificity for the enzyme, which will result in lesser adverse effects and overall, lesser toxicity. This can exclusively divert more attention towards the understanding and development of natural mixed inhibitors of PTP1B. Inhibitors such as orientin as shown in this study are classified to be selective and have been reported to oxidize the Cys215 (catalytic residue) [36], which is favorable for inhibiting PTP1B.

Several current drugs used in the management of diseases particularly T2DM are prone to adverse effects aside the inaccessibility of these agents to sufferers. Hence, plant-based drug candidates with relatively lesser or minimal side effects are explored for diabetes control. Interestingly, flavonoids, one of the vital secondary metabolites of medicinal plants, have been documented with several pharmacological potentials including antihyperglycaemic effect. In fact, flavonoids such as apigenin, rutin, luteolin, and hesperidin are established (*in vitro* and *in vivo*) as good inhibitors against numerous diabetes targets [67]. The possible identification of therapeutic potentials of orientin (comparable to ursolic acid) which was confirmed through *in silico* and *in vitro* investigation in this study presents a positive insight into further confirmatory studies on its potential in animal models and clinical studies, and efforts are underway in this direction.

## 4. Conclusion

The findings from this study established flavonoid C-glycosides as PTP1B inhibitors with orientin being the most potent *in silico* and were further evaluated *in vitro*. Orientin dose-dependently inhibited the specific activity of PTP1B in a mixed manner suggestive of selective inhibitory effect with the results of the *in vitro* analysis in agreement with those of the *in silico* evaluation. Put together, while the findings from this study strongly suggest orientin as a lead compound against PTP1B for the management of T2DM, further studies are imperative to focus on structural modifications of orientin to address the Lipinski's violations with a view to improve its druggable attributes as a novel PTP1B inhibitor. Efforts are underway in this direction.

## Figures and Tables

**Table 1:** Docking scores of complexations between flavonoid C-glycoside compounds and PTP1B.

Flavonoid C-glycoside	Binding affinity (kcal/mol)
Apigenin	-7.3
Aspalathin	-7.0
Isoorientin	-5.5
Isovitexin	-6.6
Puerarin	-6.5
Orientin	-7.3
Vitexin	-7.3
Ursolic acid	-7.4

**Table 2:** Thermodynamic binding free energy component for flavonoid C-glycosides towards PTP1B.

Energy components (kcal/mol)					
Complexes	Δ*E*_vdW_	Δ*E*_elec_	Δ*G*_gas_	Δ*G*_solv_	Δ*G*_bind_
PTP1B + ursolic acid	−23.24 ± 6.18	−31.35 ± 16.63	−54.59 ± 17.63	35.35 ± 12.90	−19.24 ± 6.95
PTP1B + vitexin	−35.32 ± 4.68	−41.38 ± 16.68	−76.70 ± 18.29	43.03 ± 12.09	−33.67 ± 7.58
PTP1B + apigenin	−31.33 ± 3.88	−18.15 ± 7.24	−49.48 ± 7.07	24.89 ± 5.11	−24.58 ± 3.59
PTP1B + orientin	−31.70 ± 3.28	−53.38 ± 7.57	−85.08 ± 7.25	50.61 ± 5.59	−34.47 ± 3.50

PTP1B: protein tyrosine phosphatase 1B; Δ*E*_vdW_: van der Waals energy; Δ*E*_elec_: electrostatic energy; Δ*E*_gas_: gas-phase free energy; Δ*G*_solv_: solvation free energy; Δ*G*_bind_: total binding free energy.

**Table 3:** Average (Å) postdynamic data of complexations of flavonoid C-glycosides and apoenzyme.

Complexes	RMSD	RMSF	SASA	ROG
PTP1B+ursolic acid	1.50 ± 0.15	1.05 ± 0.51	12613.10 ± 283.60	18.94 ± 0.09
PTP1B+vitexin	1.12 ± 0.22	0.98 ± 0.63	12307.95 ± 364.07	18.81 ± 0.08
PTP1B+ apigenin	1.70 ± 0.23	1.22 ± 0.65	13019.15 ± 336.62	18.92 ± 0.10
PTP1B+orientin	1.32 ± 0.10	0.99 ± 0.52	12621.41 ± 284.23	18.85 ± 0.06
PTP1B	1.68 ± 0.24	1.28 ± 0.87	13194.41 ± 312.84	18.92 ± 0.07

PTP1B: protein tyrosine phosphatase 1B; RMSD: root mean square deviation; RMSF: root mean square fluctuation; SASA: solvent accessible surface area; ROG: radius of gyration.

**Table 4:** Druglikeness properties of orientin and ursolic acid.

Compound	Orientin	Ursolic acid
Molecular weight	448.38 g/mol	456.70 g/mol
Number of rotatable bonds	3	1
No. of H-bond acceptor	11	3
No. of H-bond donors	8	2
TPSA	201.28 Å	57.53 Å
Log *P*	1	3.95
Water solubility (log *S*)	-2.70 (soluble)	-7.23 (poorly soluble)
Bioavailability	0.17	0.85
No. of violations	2 violations: N or O > 10 and NH or OH > 5	1 violation, Log*P* > 4.15

TPSA: topological polar surface area.

**Table 5:** Inhibitory potential (IC_50_) of orientin and ursolic acid against PTP1B.

Compound	IC_50_ (mg/ml)
Orientin	0.18 ± 0.02^a^
Ursolic acid	0.13 ± 0.02^b^

Values are expressed as mean ± standard error of the mean (SEM) of triplicate determinations. ^a,b^Values with different superscripts are significantly different (*P* < 0.05).

**Table 6:** Kinetics of inhibition of protein tyrosine phosphatase 1B by orientin.

Compound	*V*_max_ (*μ*M/s)	*K*_*m*_ (*μ*M)	*K*_cat_ (*μ*M/s)
Orientin	0.004	0.515	0.048
Control	0.005	0.048	0.018

*V*_max_: maximum velocity; *K*_*m*_: Michaelis constant; *K*_cat_: turnover number.

## Data Availability

Data used in the study are available in the manuscript.

## Supplementary Materials

Supplementary Materials The binding free energy and amino acid interactions of the docked compounds against PTP1B and their interaction plots are presented in Table S1 and Figure S1, respectively.
