# Supplementary material for: Identification of Flavonoid C-Glycosides as Promising Antidiabetics Targeting Protein Tyrosine Phosphatase 1B
Source: J Diabetes Res. 2022 Jun 24;2022:6233217. doi: 10.1155/2022/6233217 (PMC9249544; doi:10.1155/2022/6233217)
Supplement: Supplementary Materials — The binding free energy and amino acid interactions of the docked compounds against PTP1B and their interaction plots are presented in Table S1 and Figure S1, respectively. [file 6233217.f1.docx]

Table S1: Binding energy and amino acid interactions of the compounds against PTP1B

| Compound | Binding affinity/Energy (kcal/mol) | Number of Hydrogen Bonds | H-bonds interacting residues | Van Der Waal interactions | Other interacting residues |
| --- | --- | --- | --- | --- | --- |
| Vitexin | - 7.3 | 4 | Arg221  Gly220  Lys116  Tyr46 | Asp 181  Cys215  Gly218  Ile219  Val49  Asp48  Lys120  Gln262 | Arg221 (Pi-cation)  Tyr46 (Pi-pi stacked)  Ala217 (Pi-alky)  Gln266 (Unfavourable donor) |
| Orientin | - 7.3 | 4 | Arg221  Asp181  Gly220  Lys120 | Ser216  Ile219  Gly218  Gln262  Cys215  Gln266  Trp179  Pro180  Phe182  Lys116  Thr263  Asp48  Val48 | Ala217 (pi-alkyl), Arg221(pi-cation) & Tyr46 (pi-pi stacked) |
| Apigenin | - 7.3 | 3 | Asp48  Asp181  Gly220 | Gln262  Ile219  Val49 | Arg221 (Pi-cation), Tyr46 (pi-pi stacked), Ala217 (pi-alkyl) and Gln216 + Lys116 (unfavorable donor-donor) |
| Ursolic acid | -7.4 | 2 | Gly183  Tyr46 | Lys120  Ser216  Aeg221  Trp179  Gln266 | - |


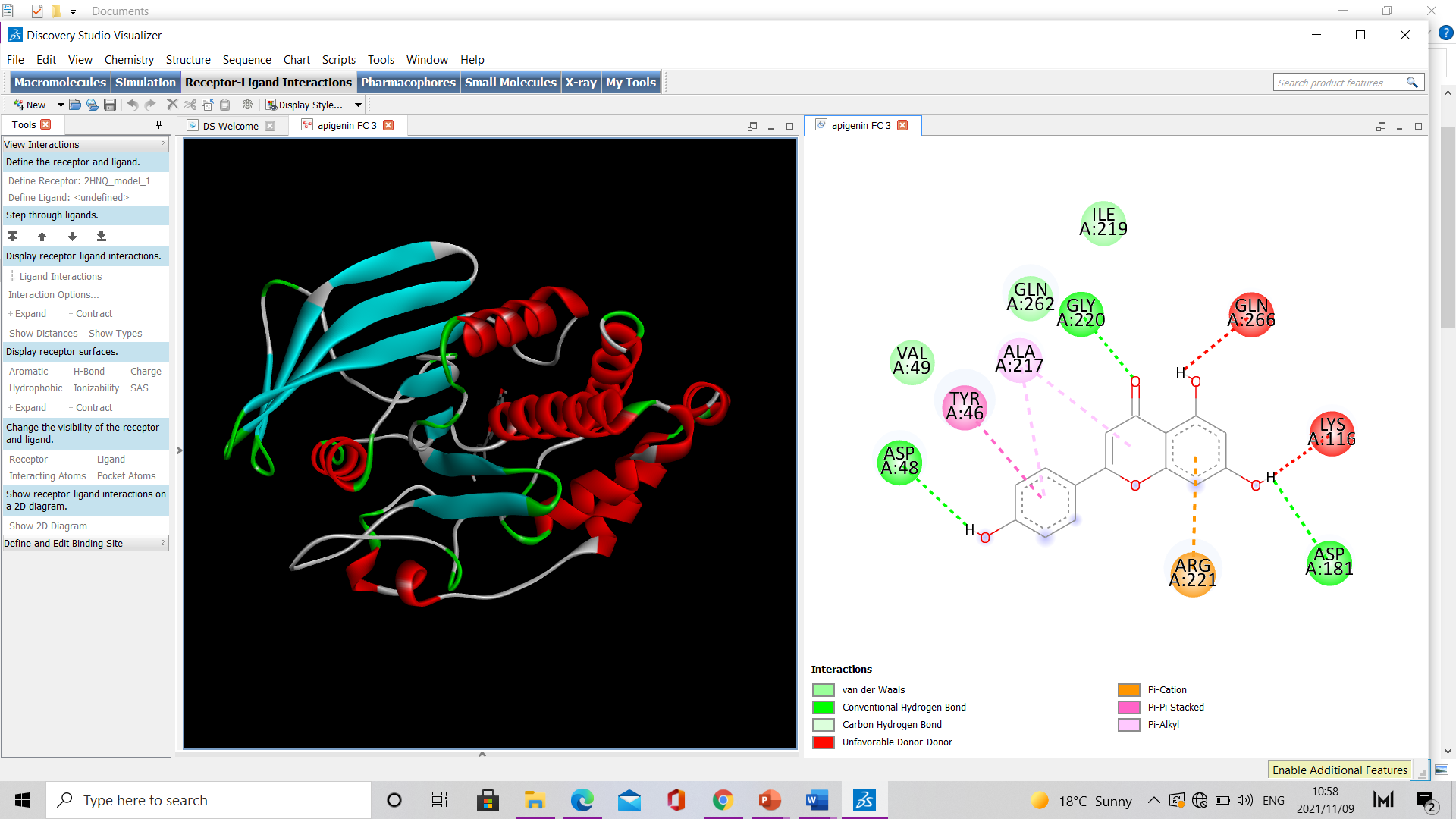
**
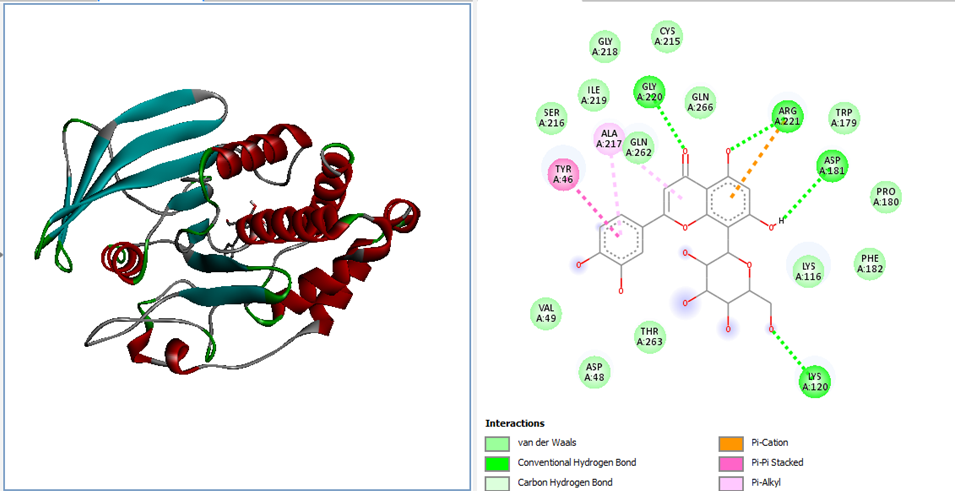
**

B)

A)

D)

C)


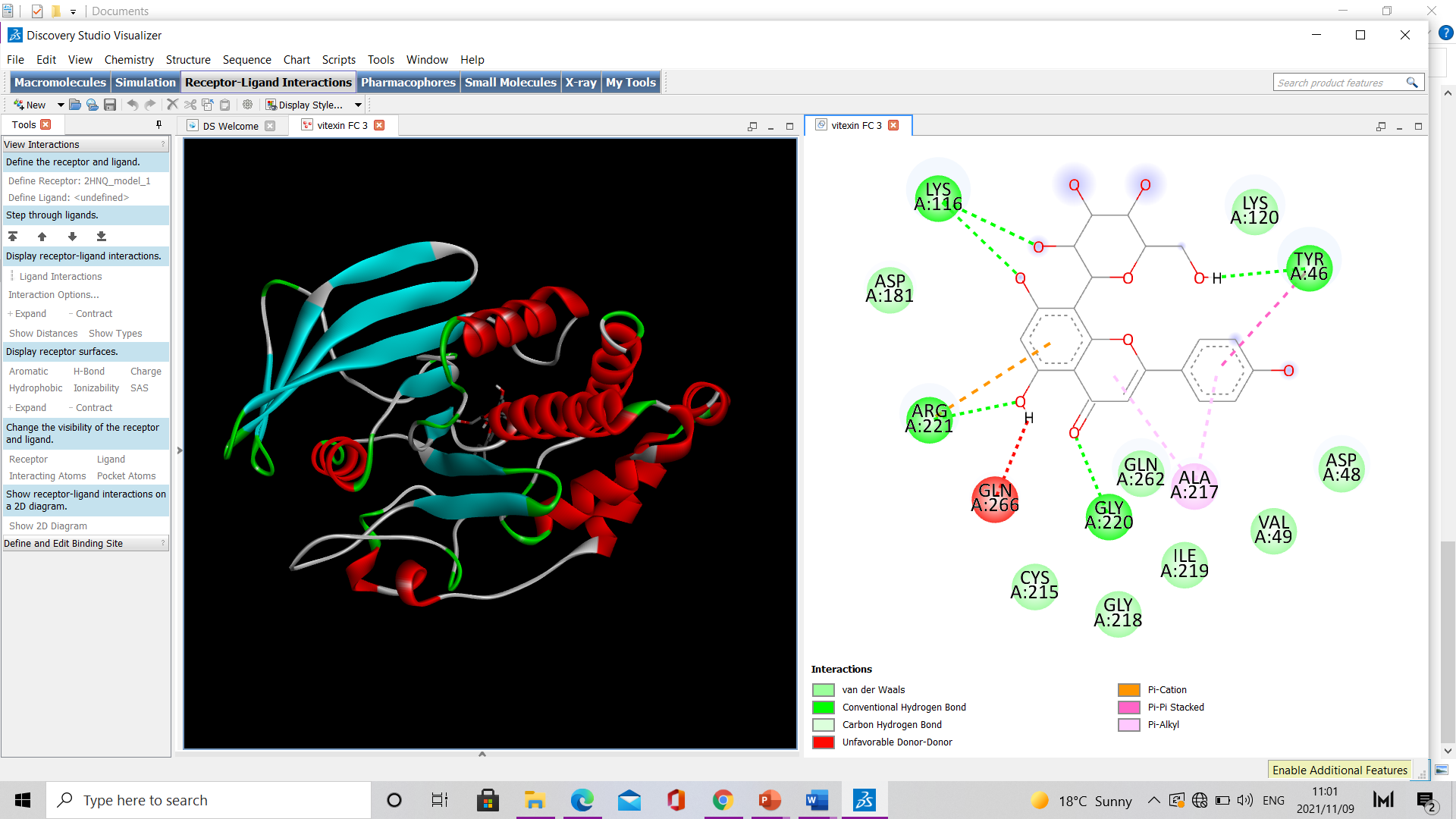

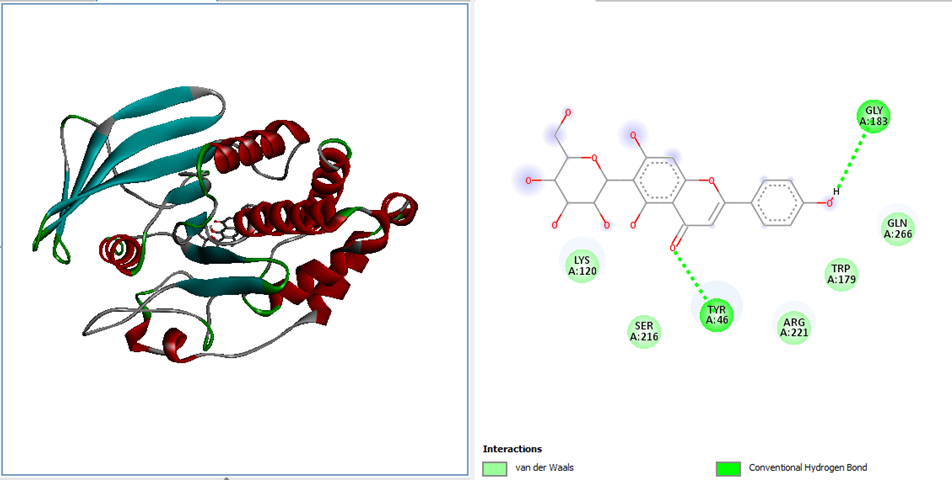


Figure S1: Interaction plots of A) Apigenin, B) orientin, C) vitexin and D) ursolic acid and PTP1B.
